# Spineless cactus (*Opuntia ficus-indica*) and saltbush (*Atriplex halimus* L.) as feed supplements for fattening Awassi male lambs: effect on digestibility, water consumption, blood metabolites, and growth performance

**DOI:** 10.1007/s11250-019-01858-6

**Published:** 2019-03-05

**Authors:** Faysal Alhanafi, Yahia Kaysi, Muhannad Muna, Ashraf Alkhtib, Jane Wamatu, Emily Burton

**Affiliations:** 1 Faculty of Agricultural Engineering, Department of Animal Production, University of Damascus, P.O Box 5735, Damascus, Syria; 2 General Commission of Scientific Agricultural Research, P.O Box 113, Damascus, Syria; 3 School of Animal, Rural and Environmental Science, Nottingham Trent University, Brackenhurst Lane, Southwell, Nottinghamshire, NG25 0QF UK; 4 International Center for Agricultural Research in the Dry Areas (ICARDA), P.O Box 5689, Addis Ababa, Ethiopia

**Keywords:** Cactus cladodes, Saltbush, Fattening, Lambs, Awassi

## Abstract

The effect of replacing 13.6% and 20.3% of a total ration of fattening Awassi lambs by two combinations of fresh saltbush (*Atriplex halimus*) and fresh spineless cactus (*Opuntia ficus-indica*) cladodes at a ratio of 1.9:1 (TRT1) and 1.7:1 (TRT2) on water intake, digestibility, blood metabolites, and fattening performance was evaluated. Thirty-six lambs with average initial live weight 34.5 ± 4.18 kg were randomly assigned to three diets (control, TRT1, and TRT2). The control received a diet containing 166 g/kg barley straw and 834 g/kg of commercial concentrate mixture; TRT1 comprised 126 g barley straw, 739 g/kg concentrate mixture, 47 g/kg spineless cactus, and 89 g saltbush; TRT2 comprised 67 g/kg barley straw, 704 g/kg commercial concentrate mixture, 86 g/kg spineless cactus, and 144 g saltbush. A growth trial of 100 days (10 days of adaptation and 90 days of collection) followed by a metabolism trial of 17 days (10 days of adaptation and 7 days of a total feces and urine collection) was carried out. Daily dry matter intake, digestibility of crude protein, ether extract and nutrient detergent fiber, nitrogen balance, average daily gain, feed conversion ratio, and blood metabolites were not significantly affected by the treatment. Water consumption in TRT2 was significantly 16% less compared with the control. A combination of saltbush and spineless cactus at a ratio of 1.7:1 (TRT2) replaced 60% of barley straw and 16% of concentrate mixture without adverse effects on health and growth performance of Awassi male lambs. This represents a potential reduction in feed costs for smallholder farmers.

## Introduction

Syria has a large flock of Awassi sheep estimated at 13.8 million heads that supplies 66% of Syria’s red meat (MOA 2016). It has been reported that more than 90% of Awassi sheep flock in Syria are raised in arid and semiarid which receive annual rainfall of less than 300 mm (Salhab and Yasin 2008). The sheep are mainly fed on natural pastures, cereal grains, and agricultural by-products (Alkhateeb 2008). Natural pastures, the basal diet of Awassi sheep in arid and semiarid areas, are continuously deteriorating in productivity and nutritive value due to deforestation (Alkhateeb 2008). Costs of cereal grains and their by-products are increasing due to the decrease in cereal yields as a consequence of drought and global climate change (Ben Salem and Smith 2008). Subsequently, feeding costs increase leading to reduced profitability of livestock production systems. In Syria, the use of alternative, cheaper, and underutilized feed options is encouraged to cope with the increasing demand of livestock feed.

Many nonconventional feeds are available for small ruminant nutrition in tropical areas (Awawdeh 2011). Feeding olive cake replaced 149 g/kg DM of the concentrate mixture without adverse effects on performance and carcass quality of Awassi fattening lambs (Abo Omar et al. 2012). Furthermore, feeding lactating sheep on crude olive cake improved fatty acid profile of milk and cheese (Vargas-Bello-Pérez et al. 2013). Incorporating dry grape pomace in diets of growing fattening sheep did not depress growth performance (Bahrami et al. 2010). Dried sugar pulp, dried citrus pulp, and olive cake can be incorporated into Awassi ewes’ diets without negative effect on milk yield and composition (Shdaifat et al. 2013). Pistachio by-products could be introduced to small ruminants’ diets at level ranging from 21 to 35%, depending on the by-product type and ruminant species, without negative effects on performance (Alkhtib et al. 2017). Inclusion of coffee pulp in growing sheep diets up to a level of 28% did not have negative effect on fattening performance (Hernández-Bautista et al. 2018).

Spineless cactus and saltbush species are reported to be suitable feed options for sheep in arid and semiarid areas. Smallholder farmers in arid and semiarid areas grow spineless cactus to produce fruits for human consumption, fences for plots and homes, and cladodes for livestock feed (Alary et al. 2007). Dry matter (DM) yield of spineless cactus varies from 3.1 to 47.3 t/ha depending on fertilization and plant density (Dubeux et al. 2006). Cladodes of spineless cactus are high in soluble carbohydrates, calcium, and vitamin A but low in crude protein (CP), fiber, and sodium (Le Houérou 1996). Supplementing straw-based diets with cladodes of spineless cactus improves ruminal digestion in sheep (Ben Salem et al. 1996). Saltbush has a high yield of edible fractions (0.5–12.3 t DM/ha), high content of CP (10–25%), high content of neutral detergent fiber (NDF) (30–45%), and moderate organic matter (OM) digestibility (460–540 g/kg) (Ben Salem et al. 2010). However, feeding sheep predominantly on spineless cactus and saltbush is associated with negative consequences on health and performance. Consuming saltbush in large amounts is associated with consumption of large quantities of water to excrete ingested salt (Ben Salem et al. 2010) whereas availability of drinking water is a critical challenge in arid and semiarid areas. Sheep fed mainly on saltbush are prone to sulfur toxicity, oxalate poisoning, and malabsorption of calcium, magnesium, and phosphorus (Ben Salem et al. 2010). High consumption of spineless cactus is expected to cause diarrhea in ruminants (Gebremariam et al. 2006). High concentration of oxalates was reported in saltbush (van Niekerk et al. 2009) and spineless cactus cladodes (Ben Salem et al. 2002b). D’Mello (1997) reported that the presence of oxalates in sheep diets at a level of 1.1 g oxalates/kg live weight is expected to result in chronic renal failure, calcium oxalate urolithiasis, hypocalcemia, and a decrease in overall performance. However, supplementation of diets based on spineless cactus with fiber-rich feeds like saltbush tends to mitigate such problems (Ben Salem et al. 2002a). As cladodes of spineless cactus contain a high level of moisture (813 to 874 g/kg DM; Batista et al. 2009), they contribute to meeting the extra demand of water resulting from feeding on saltbush. Thus, partial replacement of fattening sheep diets by a combination of fresh spineless cactus and fresh saltbush may raise productivity and decrease feeding costs of sheep in arid and semiarid areas. The current study aimed to evaluate the substitution potential of combinations of spineless cactus and saltbush in typical Syrian fattening diets of Syrian Awassi lambs comprising barley straw and concentrate mixture and their effects on voluntary DM and water intake, digestion of nutrients, nitrogen balance, blood metabolites, and growth performance.

## Materials and methods

### Animals

Animals were housed in Karahta Research Station of the General Commission of Scientific Agricultural Research, Damascus, Syria (33^○^ 4′ N, 36^○^ 5′ E) at an altitude of 616 m.a.s.l. and average rainfall of 125 mm. This study has been approved by the ethical committee of Damascus University, Syria.

Thirty-Six Awassi male lambs (34.5 ± 4.18 kg live weight and 162 ± 6 days age) were used in this trial. Lambs were housed in individual pens (2 × 1.5 m) in an open-sided barn. Each pen was equipped with a feeder and waterer. Lambs were randomly allocated into three dietary treatments with 12 repetitions. Lambs were drenched with ivermectin at rate of 200 mcg/kg live weight to control common parasites and vaccinated against common diseases of fattening sheep in Syria (anthrax, pasteurellosis, and enterotoxemia) and adapted to pens and diets for 2 weeks before the beginning of the 90-day growth trial.

### Dietary treatments

Three rations were designed with different combinations of spineless cactus and saltbush*.* The experimental diets consisted of a control and two treatment diets (TRT1, TRT2). The control consisted of 166 g/kg barley straw and 834 g/kg concentrate mixture. The concentrate mixture in the trial consisted of 500 g/kg DM whole barley grains, 270 g/kg DM whole corn grains, 170 g/kg DM cotton seed cake, 40 g/kg DM wheat bran, and 20 g/kg DM premix. No further process was applied to the concentrate mixture. This diet is commonly used by Syrian smallholders for sheep fattening. In TRT1, saltbush and spineless cactus cladodes (1.9 to 1) replaced 24% of barley straw and 11% of the concentrate mixture (on a DM basis) of the control group. In TRT2, saltbush and spineless cactus cladodes (1.7 to 1) replaced 60% of barley straw and 16% of the concentrate mixture (on a DM basis) of the control group. All rations were formulated to be isoenergetic and isonitrogenous (Table 1) formulated based on nutritional requirements for growing lambs (NRC 2007).

**Table 1:** Ingredients and chemical composition (on dry matter basis) of the experimental feeds

Diet composition (g/kg DM)	Experimental diets				
	Control	TRT1	TRT2		
Barley straw	166	126	67		
Concentrate	834	739	704		
Spineless cactus cladodes	0	47	86		
Saltbush	0	89	144		
Chemical analysis (g/kg DM)	DM	OM	CP	EE	NDF
Barley straw	92.1	93.8	3.7	0.983	80
Barley grains	91	96.1	11.8	1.9	21.7
Corn grains	90	98.1	9.1	3.9	11.9
Wheat bran	89.9	94.7	15.2	4.3	44.9
Cotton seed cake	92.4	94.2	34.5	6.4	34
Premix	99	0	0	0	0
Spineless cactus cladodes	9.9	82.5	6.1	2.2	26
Saltbush	25.2	82.2	11.7	2.3	52
Treatment					
Control	91.7	94.1	13.8	2.99	31.5
TRT1	59.3	92.8	13.5	2.93	32
TRT2	47.4	86.1	13.8	2.95	30.5

*DM*, dry matter; *CP*, crude protein; *EE*, ether extract; *NDF*, neutral detergent fiber; *OM*, organic matter; *Control*, control group; *TRT1*, saltbush and spineless cactus cladodes (1.9 to 1) replaced 24% of barley straw and 11% of the concentrate mixture of control group; *TRT2*, saltbush and spineless cactus cladodes (1.7 to 1) replaced 60% of barley straw and 16% of the concentrate mixture

### Experimental procedures

Forages of 5-year-old saltbush (*Atriplex halimus* L*.*) shrubs and a 2-year-old spineless cactus (*Opuntia ficus-indica*) grown in demonstration fields at a density of 2500 and 5000 plants/ha respectively were used. Fresh leaves and young twigs of saltbush biomass in addition to cladodes of spineless cactus were manually harvested on a daily basis during the trial. Both saltbush and spineless cactus were chopped to a theoretical size of 5 cm and fed fresh.

The lambs received a daily total DM of 4% of their live weight. Concentrate mixture and barley straw were distributed daily at 8:30 h and 17:30 h in two equal portions while saltbush and spineless cactus were offered fresh at 12:30 h. All lambs had ad libitum access to clean drinking water. Feed offered and refusals were recorded daily prior to the morning feeding to obtain daily feed intake for each lamb. Live weight of lambs was measured once every 10 days before the morning feeding to estimate daily weight gain. Blood samples were collected into two tubes on start day then monthly (4 samplings in total) before the morning feeding via the jugular vein: one containing heparin to estimate hematological parameters and the other one without heparin to obtain serum. Serum samples were obtained by centrifuging (1677×*g*; 20 min; 4 °C) of whole blood. The sera were stored at − 20 °C until being analyzed.

At the end of growth trial, 3 lambs were randomly selected from each treatment group and transferred to individual metabolic crates. After a 14-day adaptation to new conditions and diets, fecal output and urine were collected for 10 consecutive days to measure the digestibility of experimental diets. Representative samples of feed distributed to each lamb and refusals were taken daily. These were dried in a forced air oven at 60 °C for 48 h, ground to pass a 1-mm screen, and stored at room temperature for subsequent analysis. Urine was collected in bottles containing 100 ml of 10% sulfuric acid and stored at − 20 °C until analyzed. Daily fecal was recorded and a representative sample for each lamb taken and frozen at − 20 °C for subsequent analysis.

### Feed and blood sample analyses

All samples of feed, leftover feeds, and feces were dried at 105 °C overnight in a forced air oven to determine DM (AOAC 2000; method 934.01). Ash was determined by burning samples in a muffle furnace at 550 °C overnight (AOAC 2000; method 942.05). The nitrogen (N) was determined according to Kjeldahl (AOAC 2000; method 954.01) and ether extract (EE) was determined using the Soxhlet method (AOAC 2000; method 920.39). Crude protein content was calculated as N × 6.26. Neutral detergent fiber (NDF) was determined according to Van Soest et al. (1991). Neutral detergent fiber was assayed without use of an alpha amylase but with sodium sulfite and expressed without residual ash. Specific commercial kits (Katal, Belo Horizonte, MG, Brazil) and a semiautomatic analyzer (Bioplus BIO-2000, Barueri, SP, Brazil) were used to analyze serum urea by the kinetic method with the use of urease (Sampson and Baird 1979), total protein by the biuret method (Tietz 1995), albumin by the boromocresol green method (Dumas et al. 1997), alanine aminotransferase activity by the kinetic method (Huang et al. 2006), aspartate aminotransferase activity by the kinetic method (Huang et al. 2006), and glucose with the use of glucose oxidase (Barham and Trinder 1972), triglyceride (McGowan et al. 1983), cholesterol (Lie et al. 1976), calcium (Leary et al. 1992), and phosphorus (Bartels and Roijers 1975). Automated hematology analyzer (Diatron, Abacus 5, Austria) was used to determine hemoglobin and packed cell volume.

### Statistical analysis

All statistical analyses were carried out using SAS 9.1.3 (SAS 2012). The experimental unit was pen, unless otherwise specified. Probability was set at *P* ≤ 0.05. Data of the growth trial and blood parameters were analyzed using a repeated measurements design. The MIXED procure of SAS with the following model was used:

$$ {Y}_{ij}=\mu +{\mathrm{TRT}}_i+{M}_j+{\left(\mathrm{TRT}\times M\right)}_i+{\varepsilon}_{ij} $$

where *Y* is the response variable, TRT is the effect of the treatment is the effect of the measurement, TRT × *M* is the effect of the interaction between treatment and measurement, and *ε* is the residual. The subject, the variable on which repeated measurements were taken, was defined as a lamb within a treatment. The type of variance-covariance structure used was set as compound symmetry.

Data of metabolism trial was analyzed according to the following model:

$$ {Y}_{ij}=\mu +{\mathrm{TRT}}_i+{\varepsilon}_{ij} $$

where *Y* is the response variable, TRT is the effect of the treatment, and ε is the residual.

Least significant difference at 0.05 level of significance was used to separate the treatments in both models.

## Results

### Metabolism trial

Intake, digestibility, nitrogen balance, and water consumption of Awassi male lambs are shown in Table 2. Replacing diets by saltbush and spineless cactus did not reduce (*P* > 0.05) dry matter intake of Awassi sheep either in form of g/day nor g/kg^0.75^ (*P* > 0.05). Increasing levels of saltbush and spineless cactus improved (*P* < 0.05) the digestibility of CP and NDF but not DM, OM, and EE. Digestibility of CP in TRT1 and TRT2 was respectively higher than that in the control group by 4.4 points and 6.3 points (*P* < 0.05). Neutral detergent fiber digestibility in the TRT 1 and the TRT 2 was higher than that in the control by 5.4 points and 9.9 points respectively. Nitrogen intake, fecal N loss, N voided in urine, and N retention of lambs were not significantly different among the treatment groups. The consumption of water by lambs decreased (*P* < 0.05) by 0.64 L/day in TRT1 and 1.07 L/day in TRT2 compared with the control. Lambs in TRT1 and TRT2 consumed less (*P* < 0.05) water than those in the control by 0.37 L/kg DM and 0.71 L/kg DM respectively. Consumption of water by lambs decreased (*P* < 0.05) by 0.02 L/kg^0.75^ in TRT1 and 0.037 L/kg^0.75^ in TRT2 compared with the control.

**Table 2:** Effect of dietary treatments on intake, digestibility, and nitrogen balance of Awassi male lambs

	Treatments			S.E.M	*P* value
	Control	TRT1	TRT2		
Feed intake					
DM (g/d)	1542	1536	1553	6.54	0.087
DM (g/kg W^0.75^)	55	55.3	55.8	1.29	0.332
Digestibility (%)					
DM	69.5	70.3	70.6	1.48	0.5
OM	72	72.4	72.7	1.4	0.344
CP	64.1^b^	68.5^a^	70.4^a^	1.08	0.004
NDF	48.1^b^	53.5^a^	58a	2.8	0.032
EE	78.8	78.5	78.3	1.21	0.333
N balance (g/day)					
N intake	34.7	34.6	35	0.061	0.437
N excrete in urine	10.5	10.5	9	1.01	0.29
N excrete in feces	11.8	10.9	10.9	0.386	0.49
N retention (g/day)	10.4	11.2	11	1.18	0.322
N retention (% of N intake)	31.9	32.1	33.6	3.48	0.66
Water balance					
Water consumption (L/day)	6.64^a^	6.0^b^	5.57^c^	0.251	0.004
Water consumption (L/kg^0.75^)	0.237^a^	0.217ab	0.2^b^	0.011	0.007
Water consumption (L/kg DM intake)	4.3^a^	3.93^b^	3.59^c^	0.169	0.003

Means within a row with different superscript lowercase letters are significantly different (*P* < 0.05). *Control*, control group; *TRT1*, saltbush and spineless cactus cladodes (1.9 to 1) replaced 24% of barley straw and 11% of the concentrate mixture of control group; *TRT2*, saltbush and spineless cactus cladodes (1.7 to 1) replaced 60% of barley straw and 16% of the concentrate mixture of control group; *DM*, dry matter; *OM*, organic matter; *CP*, crude protein; *NDF*, neutral detergent fiber; *EE*, ether extract; *W*^*0.75*^, metabolic body weight

### Growth performance

Table 3 presents the effect of treatments on growth performance of Awassi male lambs. The difference in dry matter intake, final weight, weight gain, average daily gain, and feed conversion ratio among experimental treatments was insignificant (*P* > 0.05). There was no significant effect of the measurement nor treatment×measurement interaction on growth performance parameters (Table 3).

**Table 3:** Effect of dietary treatments on growth performance of Awassi male lambs

	Treatments			S.E.M	*P* values		
	Control	TRT1	TRT2		TRT	M	T×M
Initial body weight (kg/head)	34	34.4	35	1.24	0.656	0.076	0.37
Final body weight (kg/head)	52.1	51.9	52.2	1.37	0.555	0.339	0.0917
Body weight gain (kg/head)	18.1	17.4	17.1	0.619	0.562	0.444	0.654
Daily gain (g/head per day)	204	200	195	7.05	0.328	0.41	0.622
Dry matter intake (g/head per day)	1477	1475	1466	59.1	0.754	0.542	0.436
Feed intake/ weight gain	7.25	7.37	7.51	0.342	0.087	0.65	0.5

*Control*, control group; *TRT1*, saltbush and spineless cactus cladodes (1.9 to 1) replaced 24% of barley straw and 11% of the concentrate mixture of control group; *TRT2*, saltbush and spineless cactus cladodes (1.7 to 1) replaced 60% of barley straw and 16% of the concentrate mixture of control group; *TRT*, the effect of treatment; *M*, the effect of measurement; *T×M*, the effect of the interaction between treatment and measurement

### Blood metabolites

Table 4 shows blood metabolites of lambs in the control, TRT1, and TRT2. Levels of all blood metabolites of lambs were not different (*P* > 0.05) among treatments. All blood parameters related to protein metabolism tended to be higher than that of the control group. Concentration of glucose and triglycerides was only numerically but insignificantly higher in TRT1 and TRT2 compared with the control group. Cholesterol level of TRT1 and TRT2 tended to be less than that of the control group. Calcium and phosphorus levels were numerically higher in TRT1 and TRT2 compared with the control group. Effect of the measurement and the interaction between treatment and measurement on blood metabolites was insignificant (*P* > 0.05) (Table 4).

**Table 4:** Effect of dietary treatments on blood parameters in Awassi male lambs

	Diets			S.E.M	*P* value		
	Control	TRT1	TRT2		TRT	M	TRT×M
Protein metabolism							
Alanine transferase (IU/L)	7.7	7.98	9.67	0.966	0.43	0.067	0.364
Aspartate transferase (IU/L)	54.9	50.4	55.9	2.81	0.55	0.092	0.426
Hemoglobin	11.3	11.3	11.5	0.177	0.09	0.651	0.1
Packed cell volume	30.1	30.5	30	0.425	0.565	0.53	0.111
Urea (mg/L)	6.15	6.19	6.35	0.222	0.092	0.391	0.326
Total protein (g/L)	67.2	67.8	68.3	0.789	0.077	0.489	0.096
Albumin (g/L)	33.9	34.3	35.2	0.471	0.453	0.239	0.439
Energy metabolism							
Glucose (mg/L)	8.16	8.42	8.52	0.141	0.32	0.453	0.288
Triglycerides (mg/L)	1.94	1.95	1.99	0.141	0.665	0.546	0.391
Cholesterol (mg/L)	4.45	4.62	4.43	0.133	0.324	0.098	0.327
Minerals metabolism							
P (mg/L)	0.358	0.337	0.329	0.012	0.312	0.211	0.4
Ca (mg/L)	0.99	0.939	0.996	0.028	0.332	0.222	0.436

*Control*, control group; *TRT1*, saltbush and spineless cactus cladodes (1.9 to 1) replaced 24% of barley straw and 11% of the concentrate mixture of control group; *TRT2*, saltbush and spineless cactus cladodes (1.7 to 1) replaced 60% of barley straw and 16% of the concentrate mixture of control group; *TRT*, the effect of treatment; *M*, the effect of measurement; *T×M*, the effect of the interaction between treatment and measurement

## Discussion

Saltbush and spineless cactus have been reported to negatively impact on sheep performance if they are fed separately. However, simultaneously introducing saltbush and spineless cactus to sheep rations in the current study made no significant difference to growth performance, but improved digestibility of CP and NDF. Saltbush content of non-protein nitrogen was reported to be high (Ben Salem et al. 2010). Therefore, replacing commercial concentrate by saltbush and spineless cactus in TRT1 and TRT2 is expected to increase content of non-protein nitrogen. Cladodes of spineless cactus contain high levels of soluble carbohydrates but low levels of NDF and CP (Ben Salem et al. 2002c). The insignificant change in blood metabolites and nitrogen balance data indicates that spineless cactus in TRT1 and TRT2 supplied ruminal bacteria with sufficient quantity of readily available carbohydrates to improve the capacity of microbial bacteria to fix ammonia released from breaking down saltbush non-protein nitrogen which resulted in observed increase in CP digestibility. The reason behind increased NDF digestibility is that NDF of barley is less digestible compared with NDF of saltbush and spineless cactus as it has less lignin. Dry mater intake of lambs was not affected by the treatments. This indicates that inclusion of a combination of saltbush and spineless cactus cladodes at a ratio of 1.7:1 replaced 60% of barley straw and 16% of concentrate mixture of the control group did not compromise palatability. Previous studies have shown consuming saltbush without concurrent spineless cactus intake by sheep was associated with an increase in water consumption (Ben Salem et al. 2004). As spineless cactus cladodes are rich in water, lambs fed on diets containing cactus cladodes consumed less water compared with the control. Therefore, inclusion of a combination of saltbush and cactus in lambs’ diets in replacement of 23% of the total diet could contribute significantly to the daily requirement of water. This is of high importance to smallholder farmers in dry lands.

A high concentration of oxalates was reported in saltbush (van Niekerk et al. 2009) and spineless cactus cladodes (Ben Salem et al. 2002a). D’Mello (1997) reported that the presence of oxalates in sheep diets at a level of 1.1 g oxalates/kg live weight is expected to cause chronic renal failure, calcium oxalate urolithiasis, hypocalcemia, and a decrease in overall performance. However, hemoglobin and packed cell volume levels of Awassi lambs were similar across all experimental treatments. That means oxalates of saltbush and spineless cactus did not depress the metabolism of copper, iron, vitamin B_11_, and vitamin B_12_. Levels of albumin, alanine transferase, and aspartate transferase in TRT1 and TRT2 were similar to those in the control. This suggests that oxalates in these treatments did not have adverse effects on liver functions, which agrees with Otal et al. (2010). Concentration of urea in blood of lambs in TRT1 and TRT2 was similar to that in the control which signifies to normal renal function in lambs fed on a mixture of saltbush and spineless cactus. Levels of calcium and phosphorus in TRT1 and TRT2 were normal and not significantly different from those in the control which denotes that oxalates in TRT1 and TRT2 did not affect metabolism of calcium and phosphorus. Blood parameters of metabolism of energy and protein were similar among the experimental treatments. That means levels of oxalates in TRT1 and TRT2 did not affect metabolism of nutrients in Awassi lambs. Moreover, it suggests that all experimental groups supplied similar levels of protein and energy. This result is in line with results of the metabolism trial which showed slight differences (*P* > 0.05) among experimental treatments in terms of digestibility of nutrients. Overall, this indicates that replacement of 60% barley and 16% concentrate with a combination of saltbush and spineless cactus cladodes (1.7:1) did not raise ration content of oxalates to a toxic level. These results agree with Otal et al. (2010) who fed sheep saltbush ad libitum for 4 weeks without negative effects on blood profile. Similarly, Rekik et al. (2010) reported that feeding Barbarine sheep on 3 kg of spineless cactus per day for 60 days did not alter blood metabolites.

Growth performance of Awassi lambs was not different among control, TRT1, and TRT2. This is in line with the results of digestibility and blood metabolites which indicated similar ingestion of nutrients among treatments. Cereal grains and agro-industrial by-products are the main source of concentrates for livestock feeding in Syria (Alkhateeb 2008). The productivity of crops and, thus, availability of their by-products in developing countries including Syria are decreasing as a result of drought and climate change (Ben Salem and Smith 2008). Furthermore, deforestation is continuously degrading productivity and nutritive value of natural pastures which are the basal diet of sheep. This will not only widen the feed gap in Syria but also lead to an increase in feed costs. Rangelands that receive less than 300 mm of rain annually and are not suitable for cropping constitute 44% of the total area of Syria (MOA 2016). Awassi sheep constitute 90% of the livestock kept by pastoralists in these areas (Salhab and Yasin 2008).

Additionally, these forages grow efficiently in arid and semiarid areas. Thus, crowing saltbush and spineless cactus could be a strategic solution to feed shortage in Syria.

Producing vegetation of saltbush and cactus will be with low cost after pastures are established. Accordingly, replacement of commercial concentrates by saltbush and spineless cactus at an optimum level would decrease sheep fattening costs in Syria.

## Conclusion

This study pinpoints that a mixture of saltbush and spineless cactus (1.7 saltbush:1 spineless cactus cladodes) can be introduced to fattening sheep diets replacing 60% of barley straw and 16% of the concentrate mixture which would decrease feeding cost without any adverse effect on health and growth. Moreover, this combination provided 16% of water requirements of fattening Awassi lambs which has special advantage in arid and semiarid areas. Thus, saltbush and spineless cactus, provided that incorporated at an optimum level, might be a sustainable feed option for sheep keepers in Syria.

The high yields of saltbush and spineless cactus (0.7–6.3 t of edible DM/ha in saltbush and 3.1–47.3 t DM/ha in spineless cactus) suggest that the excess biomass should be preserved to facilitate transportation for use by farmers in peri-urban areas of the large cities. Therefore, more studies on the effect of preservation method of saltbush and spineless cactus on nutritive value are required. Awassi sheep is meat-milk-wool breed. Thus, effect of introducing saltbush and spineless cactus cladodes to Awassi sheep diets on milk and wool production and quality should be studied.
